# Proteomic Profiling of the Dystrophin-Deficient *mdx* Phenocopy of Dystrophinopathy-Associated Cardiomyopathy

**DOI:** 10.1155/2014/246195

**Published:** 2014-03-20

**Authors:** Ashling Holland, Kay Ohlendieck

**Affiliations:** Department of Biology, National University of Ireland, Maynooth, County Kildare, Ireland

## Abstract

Cardiorespiratory complications are frequent symptoms of Duchenne muscular dystrophy, a neuromuscular disorder caused by primary abnormalities in the dystrophin gene. Loss of cardiac dystrophin initially leads to changes in dystrophin-associated glycoproteins and subsequently triggers secondarily sarcolemmal disintegration, fibre necrosis, fibrosis, fatty tissue replacement, and interstitial inflammation. This results in progressive cardiac disease, which is the cause of death in a considerable number of patients afflicted with X-linked muscular dystrophy. In order to better define the molecular pathogenesis of this type of cardiomyopathy, several studies have applied mass spectrometry-based proteomics to determine proteome-wide alterations in dystrophinopathy-associated cardiomyopathy. Proteomic studies included both gel-based and label-free mass spectrometric surveys of dystrophin-deficient heart muscle from the established *mdx* animal model of dystrophinopathy. Comparative cardiac proteomics revealed novel changes in proteins associated with mitochondrial energy metabolism, glycolysis, signaling, iron binding, antibody response, fibre contraction, basal lamina stabilisation, and cytoskeletal organisation. This review summarizes the importance of studying cardiomyopathy within the field of muscular dystrophy research, outlines key features of the *mdx* heart and its suitability as a model system for studying cardiac pathogenesis, and discusses the impact of recent proteomic findings for exploring molecular and cellular aspects of cardiac abnormalities in inherited muscular dystrophies.

## 1. Introduction

Primary genetic abnormalities in the dystrophin gene result in the early-onset and debilitating muscle wasting disease Duchenne muscular dystrophy or the delayed-onset and milder disorder Becker muscular dystrophy [1–3]. In addition, mutations in cardiac dystrophin are linked to X-linked dilated cardiomyopathy in teenage men [4–6]. A variety of primary or secondary abnormalities in dystrophin-associated proteins are involved in several forms of limb-girdle muscular dystrophy, congenital muscular dystrophy, and dystroglycanopathy [7–9]. The Duchenne type of muscular dystrophy is the most frequently inherited neuromuscular disorder of childhood [10]. It occurs in approximately 1 in 3,500 live born males with substantial regional and national differences in disease frequency [11–13]. Early symptoms of muscular weakness are usually present before 5 years of age and drastically increased levels of serum creatine kinase, pyruvate kinase, and carbonic anhydrase isoform CA3 are characteristic for this type of inherited muscle disease  [14–16]. The highly progressive nature of symmetrical muscle wasting often causes a loss of unassisted ambulation around 12 years of age.

Muscle biopsies show an abnormal variation in fibre diameter, large numbers of fibres with central nucleation, necrosis, and a certain degree of regenerating fibres, as well as a progressive increase in fat and connective tissue [10, 20, 21]. In muscle biopsy specimens from Duchenne patients, dystrophin isoform Dp427 is completely or almost completely absent from contractile fibres [22]. In some cases, rare reverting mutants may account for a small percentage of dystrophin-positive muscle fibres [23]. Besides effects on skeletal muscle integrity, abnormalities in dystrophin are also linked to nonprogressive forms of mental retardation [24, 25], scoliosis [26, 27], impaired respiratory function [28, 29], and cardiomyopathic complications [30, 31]. The fact that respiratory care of Duchenne patients has greatly improved over the years gives the treatment of dystrophinopathy-associated cardiomyopathic side effects a more prominent role in the overall therapy of Duchenne muscular dystrophy [32–34].

This review briefly outlines the pathophysiological significance of cardiomyopathic complications in dystrophinopathies and then focuses on the scientific impact of recent mass spectrometry-based studies of cardiac abnormalities in X-linked muscular dystrophy. Below sections summarize the clinical cardiac symptoms of dystrophinopathy and the pathoanatomical, pathophysiological, and pathobiochemical aspects of the* mdx* mouse heart model of Duchenne muscular dystrophy. Following a brief introduction into the principles of cardiac proteomics as a major biomarker discovery tool for improving our general understanding of cardiac disease mechanisms, recent findings from gel-based proteomic analyses of dystrophin-deficient cardiac tissue and label-free mass spectrometric studies of the aging* mdx* heart are discussed. The considerable influence of cardiac proteomics on the field of muscular dystrophy research and the usefulness of newly discovered proteomic biomarkers for improving diagnostic procedures, prognosis of cardiomyopathic complications in dystrophinopathies, and the evaluation of novel pharmacological or cell-based treatment strategies is examined.

## 2. Cardiac Dystrophin-Glycoprotein Complex

For a full comprehension of the molecular and cellular complexity of dystrophinopathy, it is important to point out that dystrophin does not exist in isolation within the subsarcolemmal membrane cytoskeleton. Although its overall protein structure and sequence similarity to members of the spectrin-like superfamily of proteins suggest that it possibly forms an intertwined lattice of dystrophin molecules underneath the sarcolemma [35], the linkage to nondystrophin molecules appears to be absolutely vital for sarcolemmal integrity and proper muscle functioning [36–38]. It is well established that the full-length protein product of the dystrophin gene with an apparent molecular mass of 427 kDa forms a supramolecular protein complex at the plasmalemma of both skeletal and cardiac muscle fibres. The core element of the dystrophin-glycoprotein complex consists of the integral glycoprotein *β*-dystroglycan of 43 kDa that directly interacts on the one hand with the actin-binding protein dystrophin in the subsarcolemmal domain and on the other hand with the extracellular laminin-receptor *α*-dystroglycan [39]. This large assembly of surface proteins forms a stabilizing linkage between the basal lamina on the outside of muscle fibres and the actin membrane cytoskeleton in the inside of contractile cells [40]. In addition to the core *α*/*β*-dystroglycan complex, a large number of additional dystrophin-associated proteins exist, including sarcoglycans, sarcospan, dystrobrevins, and syntrophins [41–44].

Differences exist between the dystrophin-associated glycoprotein complex from skeletal muscle and heart with respect to subcellular localization and protein composition. While the muscle complex is highly enriched in the sarcolemma [45] and at the neuromuscular junction [46], in coexistence with the utrophin-glycoprotein complex [47], the cardiac dystrophin complex is also present in the transverse tubular system [48, 49]. The cardiac dystrophin-glycoprotein complex partially associates with costameric vinculin, suggesting a mechanical role in the maintenance of surface membrane integrity and membrane domain organization [50, 51]. Of note, the recent proteomic analysis of the cardiac dystrophin complex suggests a different range of indirectly associated proteins as compared to skeletal muscle fibres. The cardiac complex appears to lack an interaction with the signaling enzyme nNOS, has a differential composition of syntrophins and dystrobrevins, and displays additional binding partners, including Cavin-1, Ahnak-1, Cypher, and Cryab [52].

## 3. Dystrophinopathy-Associated Cardiomyopathy

Although dystrophinopathies are primarily categorised as disorders of the neuromuscular system [10], heart disease also plays a crucial role in the etiology of X-linked muscular dystrophy [53]. Almost all patients afflicted with Duchenne muscular dystrophy show clinical cardiac symptoms, especially during the second decade of life [54]. These cardiac abnormalities may include arrhythmias, cardiomyopathy, and regional wall abnormalities [55–59]. A gradual replacement of contractile cardiac fibres by noncontracting cell populations, such as connective and fatty tissue, causes a critical loss of cellular function in the heart of Duchenne patients [55]. The highly progressive decline in the cardiomyocyte population is probably closely connected to the limited regenerative capacity of dystrophin-deficient heart fibres. In contrast to dystrophic skeletal muscles, the heart does not undergo extensive cycles of fibre degeneration and regeneration in dystrophinopathy. In a large number of Duchenne cases, serious cardiac complications result in death [54], warranting special attention to the pathophysiological role of cardiac dystrophin and its associated glycoprotein complex. The primary loss of cardiac dystrophin results initially in changes in dystrophin-associated glycoproteins which in turn triggers a plethora of secondary cellular abnormalities, including sarcolemmal disintegration, necrosis, fibrosis, fatty tissue replacement, and interstitial inflammation. Cellular degeneration leads to progressive cardiac disease and thus fatal complications in Duchenne muscular dystrophy [60].

## 4. The Cardiac* mdx* Model of Dystrophinopathy

The pathological status of the* mdx* mouse model of Duchenne muscular dystrophy is based on a point mutation in exon 23 of the dystrophin gene, resulting in a truncated protein product that is quickly degraded in dystrophic fibres [61]. Interestingly, different types of muscle exhibit greatly varying degrees of tissue degeneration. While laryngeal, extraocular, and* interosseus* muscles show a relatively mild phenotype [62–64] and leg muscles such as* soleus*,* gastrocnemius*,* extensor digitalis longus, *or* tibialis anterior* are moderately weakened by segmental necrosis [65–67], the diaphragm represents the most severely disturbed skeletal muscle type [68, 69] in the* mdx* mouse. Besides the skeletal musculature, the* mdx* heart is also affected by a large number of cellular, physiological, and biochemical abnormalities, as recently discussed in several extensive reviews on the cardiac phenotype of dystrophinopathy [70–72]. Thus, if one takes into account the biological limitations of genetic mouse models as surrogates for human disorders, the* mdx* mouse can be employed as an excellent model system to study basic pathophysiological mechanisms of muscular dystrophy [73].

The dystrophin-deficient heart from* mdx* mice clearly exhibits abnormal histological features, including necrosis, fibrosis, and inflammation [74]. On the subcellular level, a considerable disorganization of the cardiac membrane surface and disruption of the transverse tubular network were revealed by scanning ion conductance microscopy [75]. Signs of overt cardiomyopathy are more pronounced in aged* mdx* mice as compared to milder cardiac alterations in young animals [76, 77]. Aged* mdx *mice showed a widespread and patchy increase in ventricular wall fibrosis [78], whereby the basal region exhibited a greater degree of fibrotic changes than the apex of the dystrophic heart [79]. The onset of fibrosis in the* mdx* heart was found to be associated with an increased expression of collagen and the connective tissue growth factor CTGF [80]. At a later stage of fibrosis, a drastic increase in connective tissue volume was accompanied by the activation of key profibrotic genes, including the heart-specific induction of the Nox4 gene [81]. Coronary endothelial cells are implicated in mediating cardiac fibrosis via transmural TGF-*β* signaling mechanisms [82]. Interestingly, physical exercise was shown to accelerate the cardiomyopathic process [83, 84]. Exercised* mdx* hearts were characterized by an increase in inflammatory cell infiltration, elevated levels of interstitial fibrosis, and a higher degree of adipose tissue deposition [83]. In the absence of the membrane cytoskeletal protein dystrophin, cardiomyocyte injury was increased considerably by workload-induced cell damage or an acute elevation of mechanical stress [85].

Histopathological features of the* mdx* heart correlate well with the assessment of functional deficits in cardiac output. The dystrophin-deficient heart showed an abnormal electrocardiogram [86] with significant tachycardia and decreased heart rate variability [87].* In vivo* cardiac MRI studies demonstrated larger right ventricular end-diastolic and end-systolic volumes and lower right ventricular ejection fractions in* mdx* mice [88]. High-resolution doppler echocardiography confirmed that the extent of changes in posterior wall thickness and left ventricular mass are dependent on the age of* mdx* mice [89]. The contractile properties of the* mdx* heart are markedly altered with a reduced force amplitude [90] and considerably prolonged half-relaxation time [91]. The pathophysiological basis of these functional abnormalities is associated with hypersensitive excitation-contraction coupling [92], increased ion fluxes through the fragile plasmalemma [93–95], elevated Ca^2+^-levels in the cytosol [96, 97], impaired cytosolic and luminal Ca^2+^-handling [98, 99], enhanced intracellular Ca^2+^-responses to mechanical challenges [97], an altered mitochondrial redox state, and an increased production of reactive oxygen species [97, 100]. Deficiency in cardiac dystrophin is postulated to cause plasmalemmal fragility, which in turn alters ion fluxes and signaling events at the surface membrane ultimately leading to a pathophysiologically elevated cytosolic Ca^2+^-concentration [101]. The Ca^2+^-dependent activation of proteolytic processes and mitochondrial dysfunction probably act as the starting point for the formation of fibrotic patches in the dystrophic heart, as recently reviewed by Shirokova and Niggli [72].

Besides dysregulation of excitation-contraction coupling and Ca^2+^-handling due to membrane perturbation, metabolic disturbances may predispose the Dp427-deficient heart to contractile dysfunction [102]. Pathobiochemically, the primary loss in cardiac dystrophin isoform Dp427 appears to affect the dystrophin-associated glycoprotein complex in a less severe way as compared to skeletal muscle, possibly due to the upregulation of the dystrophin homologue utrophin [47]. In normal heart, the cardiac-specific dystrophin-glycoprotein complex localizes to the sarcolemma and transverse tubules [48, 49, 103] and probably functions as a membrane-stabilizing linker during excitation-contraction-relaxation cycles in a similar way as the skeletal muscle complex [50, 51], although differences in its composition suggest additional functions [52]. In dystrophy-related cardiomyopathy, both the abundance and glycosylation of *α*-dystroglycan were shown to be altered in dystrophin-deficient heart muscle [104, 105]. In order to study global changes downstream from the primary defect in dystrophin and secondary alterations in the dystroglycan complex, mass spectrometry-based proteomics was employed for the large-scale analysis of the dystrophic heart.

## 5. Cardiac Proteomics

Over the last few years, mass-spectrometry-based proteomics has been widely applied to studying cardiac tissues in health and disease. A variety of extensive reviews have been published that summarize and discuss the underlying objectives of cardioproteomic strategies  [106, 107], the usefulness of proteomic biomarker research for improving diagnostic, prognostic and therapeutic approaches [108–110], the application of clinical proteomics in the study of cardiovascular diseases [111–113], the evaluation of post-translational modifications in cardiac proteins [114, 115], and technological advances in the field of mass spectrometry and cardiac proteomics [106, 116]. Mass spectrometry-based proteomics was instrumental in the cataloging of the protein constellation of normal heart tissue [117–121], the global assessment of changes in the cardiac proteome during development [122], the determination of functional adaptations following exercise [123–125], and the establishment of protein changes during the natural aging process [126–130], as well as the biomedical analysis of a variety of heart diseases in patients or animal models of heart disease, including dilated cardiomyopathy, atrial fibrillation, the diabetic heart, and cardiac failure [131–136]. The total number of proteins belonging to cardiac tissues is not known, since no one proteomic method can completely separate and accurately identify all proteins within a complex tissue that exhibits a wide dynamic concentration range. Most likely, the cardiac proteome consists of several thousand different protein species with a wide range of posttranslational modifications [117–121]. For a comprehensive analysis of changes in cardiac proteins with greatly differing physicochemical properties with respect to size, charge, and hydrophobicity, a combination of various proteomic techniques is often advantageous.

Diverse proteomic approaches and methods have been applied in global studies of the heart. For the initial large-scale separation of distinct protein populations, both gel-based and/or liquid chromatography-focused techniques have been employed. Labeling methodology or label-free applications were routinely used for the high-throughput identification of cardiac proteins. Proteomic methods that involve gel electrophoresis are highly suitable for the analysis of contractile proteins, regulatory proteins, metabolic enzymes, metabolite transporters, and molecular chaperones [118]. Two-dimensional gel electrophoresis can conveniently separate cardiac proteins in the range of approximately 10 kDa to 200 kDa and isoelectric points ranging from pH3 to pH11 [117, 118, 120]. Combinations of isoelectric focusing with narrow- or wide-range immobilised pH gradients, native gel electrophoresis, nonreducing gel electrophoresis, and reducing gel electrophoresis can be used for various two-dimensional applications [137–140]. While post-electrophoretic staining with protein dyes is relatively cheap and fast, the differential pre-electrophoretic labeling with fluorescent CyDyes usually results in a larger number of identified cardiac proteins and greatly reduces gel-to-gel variations [141, 142]. One-dimensional gradient gels, in combination with on-membrane digestion protocols, can also cover the separation of high-molecular-mass proteins following detergent solubilization [143]. However, low-abundance proteins, hydrophobic proteins, and components with extreme p*I*-values are difficult to study using routine gel electrophoretic methods [137, 140].

The usefulness of alternative gel-free proteomic labeling methods, such as iTRAQ (isobaric tags for relative and absolute quantitation) or SILAC (stable isotope labeling by amino acids in cell culture), which have been successfully applied to studying cardiac cells [144, 145], has been described in recent reviews [106, 107]. One of the most advanced proteomic approaches involves label-free mass spectrometry. The advantages of this method are that it (i) requires only very small amounts of protein samples, (ii) has broad applicability, (iii) detects a large range of cardiac protein species, and, most importantly, (iv) does not require protein labeling [146]. Thus, in order to overcome some of the problems associated with gel-based methods in cardiac proteomics, label-free mass spectrometry has recently been applied to investigate cardiomyopathic tissue from the aged* mdx* model of Duchenne muscular dystrophy [18].

**Figure 1:**
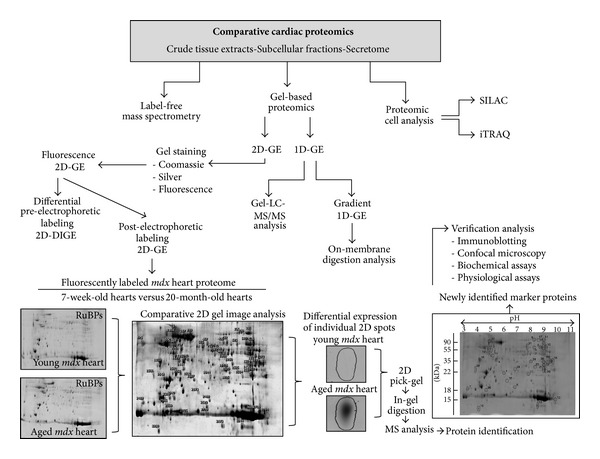
Overview of proteomic methods used in comparative studies of the heart. Shown is a flowchart of the various techniques used to identify changes in the cardiac proteome, including label-free mass spectrometry, gel-based methods (GE, gel electrophoresis), and cellular analyses (SILAC, stable isotope labeling by amino acids in cell culture; iTRAQ, isobaric tags for relative and absolute quantitation). To illustrate the typical work flow of a gel-based analysis of the dystrophic heart proteome, two-dimensional gels representing the urea-soluble proteome from the young versus the aged* mdx* heart are shown. The post-electrophoretic labeling of cardiac proteins with the fluorescent dye RuBPs (ruthenium II tris bathophenantroline disulfonate) was carried out by standard methodology [17].

Figure 1 gives an overview of the key methods employed in comparative cardioproteomic studies and illustrates typical findings from a gel-based analysis of the dystrophic heart proteome. Shown are two-dimensional gels representing the urea-soluble proteome from the young versus the aged* mdx* heart, post-electrophoretically labeled with the fluorescent dye RuBPs (ruthenium II tris bathophenantroline disulfonate) [147]. Fluorescent labeling with RuBPs dye is an excellent and cheap alternative to the more labor-intensive 2D-DIGE approach with its relatively expensive CyDyes [148]. The individual analytical steps performed to achieve the two-dimensional gel image depicted in Figure 1 have been previously described in detail by our laboratory [17].

## 6. Gel-Based Analysis of Cardiac Changes in Dystrophinopathy

Prior to the development of the proteomic concept and the streamlining of established biochemical techniques for the large-scale analysis of entire protein populations, protein biochemical studies of the dystrophic* mdx* heart have mostly focused on individual proteins, protein complexes, specific pathways, or signalling cascades. Such focused protein chemical approaches, also highly informative about specific aspects of a disease process, inevitably generate biomedical data sets with limited scope. Hence, in order to better complement findings from detailed physiological, cell biological, and histological studies of cardiomyopathic changes, mass spectrometry-based proteomics was used to establish proteome-wide alterations in* mdx *preparations. The parallel analysis of hundreds of cardiac proteins promised to swiftly determine their molecular fate in dystrophin-deficient heart tissues and thus decisively improve our understanding of the molecular pathogenesis of dystrophy-associated cardiomyopathy. Initially, comparative proteomic studies used gel-based surveys of the* mdx* heart muscle and revealed novel changes in proteins mostly associated with mitochondrial energy metabolism, the contractile apparatus, the cytoskeleton, and the cellular stress response [141, 142]. Both studies used fluorescence two-dimensional difference in-gel electrophoresis (2D-DIGE) for the analysis of the dystrophic heart.

The 2D-DIGE technique is an extremely powerful preelectrophoretic labeling approach that can swiftly determine potential changes in the concentration of thousands of proteins in large analytical gel systems [149–151] and has proven to be an excellent biomarker discovery tool for comparative studies of contractile fibres [152]. The 2D-DIGE method has been widely applied to studying various subtypes of muscle in animal models of Duchenne muscular dystrophy [153–158]. It is one of the key techniques in comparative gel-based proteomics and is employed with fluorescent 2-CyDye [159] or 3-CyDye [160] labeling systems for the differential tagging of proteins from dissimilar mixtures prior to two-dimensional gel electrophoresis [149]. The optimized analysis of 2D-DIGE images with advanced 2D software analysis tools [161–163] can highly accurately quantitate multiple protein samples on the same two-dimensional gel [164, 165]. Importantly, the completion of reverse DIGE labeling controls is not usually necessary, since selective labeling artifacts were shown not to play a significant role in the analysis of soluble proteins [166], which considerably lowers the overall time and costs involved in large-scale 2D-DIGE studies. The analysis of the murine heart proteome with the 2-CyDye labeling system and the combination of pH 4–7 and pH 6–11 gels resulted in the identification of 2,509 distinct protein spots [142], illustrating the powerful separation and labeling capabilities of the 2D-DIGE technique within the field of gel-based comparative cardiac proteomics [106].

The proteomic profiling of 1-to-9-month-old* mdx* heart extracts by Gulston et al. [141] revealed differential expression patterns for ATP synthase, glyceraldehyde-3-phosphate dehydrogenase, serine proteinase inhibitor, trifunctional enzyme, and hemoglobin. Additional metabolomic analyses suggest metabolic disturbances in the dystrophic heart, agreeing with the altered concentration of key mitochondrial and glycolytic enzymes [141]. Since abnormal heart function was shown to be prominent at 9 months of age [81], a detailed 2D-DIGE analysis of potential changes in the concentration of distinct proteins was carried out with cardiac proteins at this age of* mdx* mice [142]. Electrospray ionization MS/MS analysis identified 26 proteins with a decreased abundance, including various myosin light chains, tropomyosin, actin, adenylate kinase, creatine kinase, vimentin, fatty acid binding protein isoform FABP3, isocitrate dehydrogenase, NADH dehydrogenase, myozenin, porin, and peroxiredoxin. In contrast, 3 heart-associated proteins were found to be significantly increased, including lamin and nucleoside diphosphate kinase. An independent verification of the DIGE analysis was performed by immunoblotting and confocal microscopy of a select group of cardiac proteins. The comparative immunoblot analysis showed a drastic decrease in the enzyme adenylate kinase, the fatty acid binding protein FABP3, isocitrate dehydrogenase, and mitochondrial porin in 9-month-old* mdx* heart tissue [142]. The decreased abundance of the AK1 isoform of adenylate kinase did not correspond with a previous combined metabolomic and proteomic analysis of the* mdx* heart [141] but agrees with several comprehensive proteomic surveys of dystrophin-deficient muscle preparations [152, 153, 167–169]. Since the proteomic result was independently confirmed by immunoblotting, it appears that cardiac nucleotide metabolism that involves adenylate kinase and creatine kinase is perturbed in the dystrophin-deficient heart.

Mitochondrial dysfunction and accompanied oxidative stress have been linked to various cardiac pathologies, including cardiomyopathy, congestive heart failure, and ischaemia reperfusion injury [170], conveying considerable importance to the results from the proteomic profiling of the* mdx* heart with respect to explaining abnormal mitochondrial function in dystrophy-associated cardiomyopathy [97]. The mitochondrial proteome from heart tissue has been well catalogued and studied using proteomic techniques, focusing especially on the role of mitochondrial proteins in bioenergetics, pathology, and the natural aging process [171–173]. The proteomic finding that a variety of mitochondrial proteins exhibit an altered concentration in the* mdx* heart [141, 142] necessitated microscopical studies in order to evaluate whether these protein alterations were due to a reduced number of organelles in cardiomyopathic tissue or based on internal changes within the mitochondrial proteome. A microscopical survey using the fluorescent labeling of mitochondria with the MitoTracker dye CMXRos, staining of nuclei with the DNA binding dye DAPI, and immunofluorescence staining of cardiac marker proteins revealed no statistically significant differences in mitochondrial content, the number of nuclei, and the subcellular localization of key mitochondrial enzymes between normal and dystrophic heart [142]. Thus, the overall isoform complement of mitochondrial enzymes is not majorly altered, but certain subspecies of distinct cardiac protein isoforms are changed due to the deficiency in dystrophin. Since cardiac mitochondria are the primary site for energy generation via oxidative phosphorylation, even subtle changes in the protein population responsible for oxidative phosphorylation complexes, the citric acid cycle, and metabolite transport can be assumed to have an extensive effect on the bioenergetic status of the* mdx* heart. Besides energy metabolism, cardiac mitochondria are also involved in calcium signaling, the regulation of apoptosis, cell cycle progression, and the production of heme and iron-sulfur clusters [170]. Therefore, alterations in the mitochondrial proteome may affect these crucial cellular functions and render the* mdx* heart more susceptible to damage pathways and ultimately to extensive fibrosis.

## 7. Label-Free MS Analysis of Cardiac Changes in Dystrophinopathy

Based on the above outlined findings from gel-based proteomic analyses of the dystrophic heart, it was concluded that changes in proteins involved in fibre contraction, nucleotide metabolism, the cellular stress response, mitochondrial bioenergetics, and fatty acid transportation play a central role in the progressive loss of cardiac function in the* mdx* model of Duchenne muscular dystrophy [141, 142]. However, since two-dimensional gel electrophoresis does not properly display very large proteins, these analyses did not produce any information on a key member of the wider network of the cardiac dystrophin-glycoprotein complex, namely, the basal lamina protein laminin. In skeletal muscle, the concentration of laminin is unexpectedly not altered in dystrophin-deficient fibres [40, 152, 174], so it was of considerable interest to determine its molecular fate in cardiac tissue and evaluate whether differences exist in the extracellular matrix of both types of contractile* mdx* tissues. Label-free mass spectrometry suggested itself as an ideal analytical way to study high-molecular-mass cardiac proteins and was therefore applied to determine global downstream effects due to dystrophin deficiency within the cardiac system.

Prior to the proteomic profiling of age-related changes in the* mdx* heart, a label-free LC-MS/MS analysis of 7-week-old dystrophic versus age-matched normal mice was carried out to initially establish potential differences between unaffected and dystrophic heart tissue at an age prior to the occurrence of extensive cardiomyopathic changes [18]. Comparative proteomics established moderate changes in 20 cardiac proteins, which clearly agrees with the relatively mild pathological phenotype in young* mdx* mice. A differential expression pattern was shown for various mitochondrial enzymes, including succinyl-CoA ligase, methylmalonate-semialdehyde dehydrogenase, 3-hydroxyacyl-CoA dehydrogenase, 2,4-dienoyl-CoA reductase, 3-ketoacyl-CoA thiolase, glutamate dehydrogenase, succinyl-CoA: 3-ketoacid-coenzyme A transferase, 2-oxoglutarate dehydrogenase, and isocitrate dehydrogenase.

**Figure 2:**
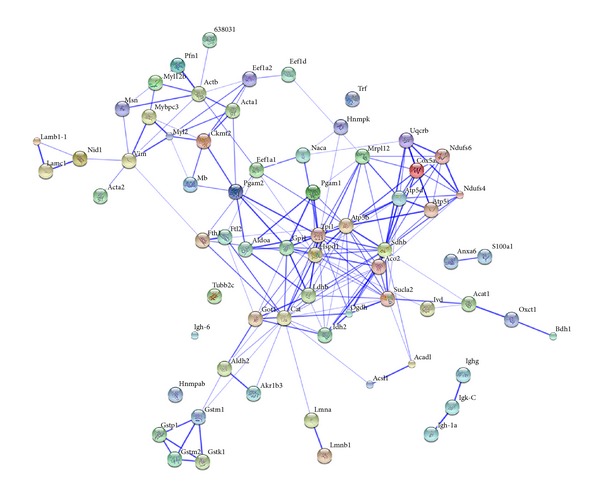
Bioinformatic STRING analysis of the proteomic data from the label-free mass spectrometric study of the aged* mdx* heart. For the evaluation of protein-protein interactions of the mass spectrometrically identified proteins with a changed abundance in the dystrophic* mdx* heart [18], bioinformatic analysis was carried out with the publically available STRING (http:// http://string-db.org/; version 9.1) database of known and predicted protein interactions that include direct physical and indirect functional protein associations [19]. The interaction map of cardiac proteins with a changed abundance in the dystrophic* mdx* heart illustrates the enormous complexity of potential protein interactions, especially with respect to mitochondrial components.

The detailed proteomic profiling of the aging process in 7-week-old to 20-month-old* mdx* hearts by label-free mass spectrometry demonstrated that aged dystrophic hearts exhibit a generally perturbed expression pattern of key cardiac proteins involved in the stabilization of the basal lamina, the organization of the cytoskeletal network, cellular iron homeostasis, antibody response, fibre contraction, and energy metabolism [18]. Age-related changes were found in 67 cardiac protein species, of which 39 proteins were shown to be increased and 28 proteins were identified as being decreased in their concentration. Of note, the most drastic alterations were increases in transferrin and various immunoglobulin chains and decreases in laminin, nidogen, and annexin. Thus, the collapse of the dystrophin network in the heart and resulting sarcolemmal fragility appears to trigger serious secondary alterations, including the disintegration of the basal lamina structure and cytoskeletal network, an increased level of antibodies in a potential autoimmune reaction of the degenerating heart, and the compensatory binding of excess iron in dystrophinopathy-related cardiomyopathy.  Figure 2 shows the bioinformatic STRING analysis of the proteomic data from the recent label-free mass spectrometric study of the aging* mdx* heart. For the evaluation of protein-protein interactions of the mass spectrometrically identified proteins with a changed abundance in the dystrophic* mdx* heart, bioinformatic analysis was carried out with the publically available STRING (http://string-db.org/; version 9.1) database of known and predicted protein interactions that include direct physical and indirect functional protein associations [19]. The interaction map illustrates the enormous complexity of potential protein interactions, especially with respect to mitochondrial components.

Functional analyses, confocal microscopy, and/or immunoblotting are routinely used to independently verify proteomic data. A comprehensive immunoblot analysis of young and senescent wild type versus* mdx* hearts has verified key proteomic results and clarified differences in protein changes due to natural aging versus muscular dystrophy [18]. While antibody decoration demonstrated that the concentration of laminin, nidogen, and annexin increased during the natural aging process, a drastic decrease in the expression levels of these 3 cardiac proteins was observed in the aged dystrophin-deficient heart. Both, the proteomic data and their confirmation by immunoblotting strongly suggest that the maintenance and architecture of the extracellular matrix, basement membrane, and cytoskeletal network are severely impaired in the aged* mdx* heart. The loss of cardiac dystrophin seems to indirectly affect the essential laminin component of the basement membrane [175] via alterations in the dystroglycan subcomplex. The reduced levels of laminin in turn appear to lower the concentration of nidogen, a sulfated glycoprotein present in many specialized basement membranes [176] and annexin, which is crucial for the maintenance of the cytoskeleton and the extracellular matrix, as well as cardiac Ca^2+^-homeostasis [177]. This loss in surface integrity of the dystrophin-deficient heart could be one of the major triggering factors that induce progressive fibrosis in dystrophinopathy-associated cardiomyopathy.

**Figure 3:**
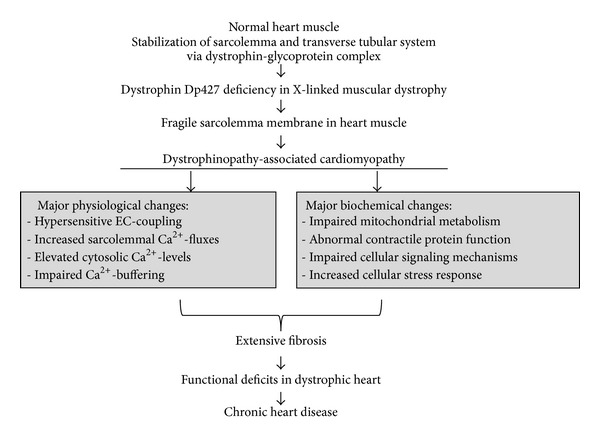
Molecular pathogenesis of muscular dystrophy-associated cardiomyopathy. Shown is a flowchart of major pathophysiological and pathobiochemical changes that render the dystrophin-deficient heart more susceptible to fibre degeneration and fibrosis, which eventually triggers chronic heart disease in dystrophinopathy. Key changes in the physiological regulation of the dystrophic heart are associated with abnormal calcium handling and hypersensitive excitation-contraction (EC) coupling.

The main findings from recent proteomic studies that have focused on the cardiac dystrophin-glycoprotein complex and dystrophin-deficient* mdx* heart tissues are listed in Table 1. The overall emphasis of the individual studies, the main technological approach, and major findings with respect to novel proteomic biomarker candidates of dystrophinopathy-associated cardiomyopathy are displayed. In addition, the flowchart in Figure 3 summarizes the variety of biochemical, physiological, and cellular abnormalities that result in cardiac fibrosis and progressive functional decline of the cardiovascular system.

## 8. Conclusions

Heart disease is a common clinical manifestation of X-linked muscular dystrophies. Hence, future approaches to treating the overall medical complications present in dystrophinopathy have to take into account the remodeling of incapacitating cardiac fibrosis and resulting functional abnormalities in the dystrophin-deficient heart. As recently reported by Wasala et al. [178], the exclusive correction of abnormalities in the dystrophic skeletal musculature unfortunately does not modulate cardiac pathogenesis in the aged* mdx* model of Duchenne cardiomyopathy. To address this biomedical issue and the fact that a high frequency of cardiomyopathy exists in teenage patients suffering from inherited X-linked muscular dystrophy, a large and diverse number of novel therapeutic approaches are currently tested to specifically address cardiac symptoms in dystrophinopathy. This includes various forms of gene therapy [179–182], exon-skipping therapy [183], and a large number of experimental drug treatments [184–192]. This in turn makes the availability of both a substantial array of reliable proteomic biomarkers and established animal models of muscular dystrophy an important prerequisite for the high-throughput and large-scale testing of new therapeutic options. In order to evaluate the long-term usefulness and potential cytotoxic side effects of gene therapy, exon-skipping, stem cell therapy, and/or pharmacological interventions, simple, cost-effective, and reliable assays with significant protein biomarkers are needed [193].

As outlined in this review, mass spectrometry-based proteomic profiling studies have clearly established the* mdx* mouse as a suitable animal model for exploring molecular and cellular aspects of cardiac pathogenesis and the aged* mdx* heart as a highly appropriate organ system for studying the progressive aspects of muscular dystrophy-associated cardiomyopathy. Most importantly, the application of comparative proteomics has identified a large number of new changes in cardiac proteins associated with cellular signaling mechanisms, mitochondrial energy metabolism, glycolysis, antibody response, iron binding, the contraction-relaxation cycle, basal lamina stabilisation, and cytoskeletal organisation. These novel protein marker candidates can now be used for the systematic screening of the cardiac* mdx* heart following experimental therapeutic interventions. The combined utilization of both label-free mass spectrometry and gel-based techniques promises the most comprehensive coverage of the cardiac proteome, including highly hydrophobic components, low-abundance elements, proteins with extreme isoelectric points, and proteins with extensive posttranslational modifications.

## Figures and Tables

**Table 1:** Proteomic profiling of the dystrophin-deficient *mdx* heart.

Proteomic study	Methods	Major findings	References
Proteomic analysis of the cardiac-specific dystrophin complex	IP-based copurification, LC-MS/MS, IB, CM	Confirmation of main dystrophin-associated proteins: dystroglycans, sarcoglycans, dystrobrevins, sarcospan, and syntrophins; plus identification of novel dystrophin-associated proteins: Cavin-1, Ahnak-1, Cypher, and Cryab	Johnson et al., 2012 [52]
Comparative proteomic study of 1-month to 9-month-old *mdx* hearts versus age-matched normal hearts	2D-DIGE, LC-MS/MS	Differential expression of ATP synthase, serine proteinase inhibitor, glyceraldehyde-3-phosphate dehydrogenase, trifunctional enzyme, and hemoglobin	Gulston et al., 2008 [141]
Comparative proteomic analysis of 9-month-old *mdx* hearts versus age-matched normal hearts	2D-DIGE, LC-MS/MS, IB, CM	Increased levels of lamin and nucleoside diphosphate kinase; drastic decrease in myosin light chains, tropomyosin, actin, adenylate kinase, creatine kinase, vimentin, fatty acid binding protein FABP3, isocitrate dehydrogenase, NADH dehydrogenase, myozenin, porin, and peroxiredoxin.	Lewis et al., 2010 [142]
Comparative proteomic analysis of 7-week-old *mdx* hearts versus age-matched normal hearts	Label-free MS analysis, IB	Moderate changes in young *mdx* hearts: actin, biglycan, troponin, protein disulphide isomerase, succinyl-CoA ligase	Holland et al., 2013 [18]
Proteomic analysis of the aging process in 7-week to 20-month-old *mdx* hearts	Label-free MS analysis, IB	Severe changes in aged *mdx* hearts: drastic reduction in laminin, nidogen, annexin, vimentin, ATP synthase, cytochromes, NADH dehydrogenase; increases in various IgG molecules, hydroxybutyrate dehydrogenase, ferritin, transferrin, catalase, glutathione transferase	Holland et al., 2013 [18]

Listed are major findings from recent proteomic studies that have focused on the cardiac dystrophin-glycoprotein complex and dystrophin-deficient *mdx* heart tissues. Abbreviations used: 2D-DIGE: two-dimensional difference in-gel electrophoresis; CM: confocal microscopy; IB: immunoblotting; IP: immunoprecipitation; LC: liquid chromatography; MS: mass spectrometry.
